# Decreased creatine kinase is linked to diastolic dysfunction in rats with right heart failure induced by pulmonary artery hypertension

**DOI:** 10.1016/j.yjmcc.2015.06.016

**Published:** 2015-09

**Authors:** Ewan D. Fowler, David Benoist, Mark J. Drinkhill, Rachel Stones, Michiel Helmes, Rob C.I. Wüst, Ger J.M. Stienen, Derek S. Steele, Ed White

**Affiliations:** a Multidisciplinary Cardiovascular Research Centre, University of Leeds, UK; b L'Institut de Rythmologie et Modélisation Cardiaque, Inserm U-1045, Université de Bordeaux, France; c Department of Physiology, Institute for Cardiovascular Research, VU University Medical Centre, Amsterdam, The Netherlands; d IonOptix LLC, Milton, MA, USA; e Department of Physics and Astronomy, Faculty of Science, VU University, Amsterdam, The Netherlands

**Keywords:** Right ventricular failure, Creatine kinase, Diastolic sarcomere length, Pulmonary artery hypertension, Monocrotaline

## Abstract

Our objective was to investigate the role of creatine kinase in the contractile dysfunction of right ventricular failure caused by pulmonary artery hypertension. Pulmonary artery hypertension and right ventricular failure were induced in rats by monocrotaline and compared to saline-injected control animals. *In vivo* right ventricular diastolic pressure–volume relationships were measured in anesthetized animals; diastolic force–length relationships in single enzymatically dissociated myocytes and myocardial creatine kinase levels by Western blot. We observed diastolic dysfunction in right ventricular failure indicated by significantly steeper diastolic pressure–volume relationships *in vivo* and diastolic force–length relationships in single myocytes. There was a significant reduction in creatine kinase protein expression in failing right ventricle. Dysfunction also manifested as a shorter diastolic sarcomere length in failing myocytes. This was associated with a Ca^2 +^-independent mechanism that was sensitive to cross-bridge cycling inhibition. In saponin-skinned failing myocytes, addition of exogenous creatine kinase significantly lengthened sarcomeres, while in intact healthy myocytes, inhibition of creatine kinase significantly shortened sarcomeres. Creatine kinase inhibition also changed the relatively flat contraction amplitude–stimulation frequency relationship of healthy myocytes into a steeply negative, failing phenotype. Decreased creatine kinase expression leads to diastolic dysfunction. We propose that this is *via* local reduction in ATP:ADP ratio and thus to Ca^2 +^-independent force production and diastolic sarcomere shortening. Creatine kinase inhibition also mimics a definitive characteristic of heart failure, the inability to respond to increased demand. Novel therapies for pulmonary artery hypertension are needed. Our data suggest that cardiac energetics would be a potential ventricular therapeutic target.

## Highlights

• Rats with pulmonary artery hypertension had right heart diastolic dysfunction.
• Failing myocytes had decreased levels of creatine kinase protein
• In failing myocytes exogenous creatine kinase reversed dysfunction.
• In healthy myocytes inhibition of creatine kinase mimicked dysfunction.
• Decreased creatine kinase is linked to dysfunction in pulmonary artery hypertension.

## 1 Introduction

Impaired systolic and/or diastolic function and a decreased ability to respond to increased demand are characteristics of the failing myocardium [1]. In tandem with these functional deficits are metabolic changes that result in compromised energy supply which may underlie the impaired function [2,3].

Creatine kinase (CK) buffers locally accumulated ADP and provides a feedback to mitochondrial respiration. It has been demonstrated, in humans with left ventricular (LV) hypertrophy, that total CK activity is decreased [4] and that CK flux is a predictor of mortality in heart failure patients [5]. At the level of the sarcomere, the myocardial contractile unit, binding of CK to the M-line has been shown to be important in maintaining normal ATP:ADP ratio, whereas depletion or inhibition of CK can lead to a local decrease in [ATP] and build-up of ADP; conditions that can favor Ca^2 +^-independent force generation [6,7].

The role of metabolic dysfunction in right ventricular (RV) failure caused by pulmonary artery hypertension (PAH) is less well studied than in left ventricular (LV) failure. Treatment of PAH is focused on the pulmonary vasculature, however, hypertension increases afterload on the RV and results in RV hypertrophy and failure. Right heart failure is the most common cause of death in patients with PAH [8,9]. There is currently no cure for PAH and an acknowledged need for novel approaches to treatment and cure [9,10]. In this context we aimed to study the possible link between CK and contractile dysfunction in a well-established animal model of PAH and RV failure.

## 2 Materials and methods

For detailed methods see Supplementary data.

### 2.1 Animal model

Male Wistar rats (200 g) received a single intraperitoneal injection of 60 mg/kg MCT to induce RV failure (FAIL) or an equivalent volume of saline as control (CON). Rats were killed upon showing external clinical signs of heart failure as previously reported (*e.g.* [11–15]). These were weight loss of at least 10 g on consecutive days, dyspnea, cold extremities and lethargy (median time 23 days, range 18–26 days) or on equivalent days for CON animals. Experiments were conducted in accord with Health Research Extension Act (public law 99–158, 1985 “Animals in Research”) and with the Directive 2010/63/EU of the European Parliament UK Home Office regulations and local ethical approval.

### 2.2 *In vivo* hemodynamics

*In vivo* hemodynamic measurements were made in mechanically ventilated rats, anesthetized with 1.5% isoflurane mixed with O_2_. The chest was opened and a Millar conductance catheter passed through the RV free wall into the RV cavity to simultaneously measure pressure and volume. Pressure–volume (PV) loops were recorded and the slope of the end-diastolic pressure–volume relationship (EDPVR) measured by occlusion of the ascending vena cava. In isolated hearts, collagen was assessed by picrosirius red staining of 10 μm thick cryosections cut through the ventricular short axis. Staining was measured by a blinded researcher using ImageJ (National Institute of Health, Bethesda, USA).

### 2.3 Single myocyte studies

Single myocytes were isolated and intracellular Ca^2 +^ ([Ca^2 +^]i) monitored using the ratiometric Ca^2 +^ dye fura-2-AM (Invitrogen, Paisley, UK) as previously described [16]. Simultaneous recordings of sarcomere length (SL) and fura-2 signal were acquired using Ion Wizard software (IonOptix, Milton, USA). Myocytes were field stimulated *via* external platinum electrodes at the desired frequency. Unless otherwise stated, experiments were carried out at 37 °C. The method of Hongo et al. [17] was used to measure myofilament Ca^2 +^ sensitivity in intact myocytes.

To investigate the control of resting SL, Tyrode solution was modified by inclusion of the intracellular Ca^2 +^ buffer BAPTA-AM (100 μM) or BAPTA-AM plus the myosin ATP-ase and cross-bridge cycling inhibitor 2,3-butanedione monoxime (BDM; 40 mM, Sigma). To inhibit all forms of CK, the irreversible agent 2, 4-dinitro-1-fluorobenzene (DNFB; 20 μM, Sigma) was added for 10 min. To test the role of CK in controlling resting SL, myocytes were permeabiliized with saponin (0.01 mg/ml) in an intracellular-like solution. Cells were incubated in this solution with or without exogenous muscle isoform CK-(MM) from bovine heart muscle (Sigma) at a concentration of 4.4 mg/ml (total activity 250 U/ml) for 30 min before SL was measured at 20–23 °C.

Mechanical manipulation of myocytes was performed with a MyoStretcher (IonOptix, Milton, USA). Myocytes were attached to stiff glass fibers using MyoTak™ glue (IonOptix) [18,19]. Cells were stretched between 2.5 and 10 μm while isometric force transients were recorded and the end diastolic force–length relationship (EDFLR), the cellular equivalent of the *in vivo* EDPVR, was measured [19].

### 2.4 Western blot analysis of creatine kinase

Muscle CK protein (CK-M) levels from RV and LV tissue isolated from CON and FAIL animals were assessed by bicinchoninic acid assay and Western blotting with primary antibodies for CK-M ((MM-2) sc-69848, 1:500, Santa Cruz Biotech and anti-GAPDH, G9545, 1:100,000, Sigma). For mitochondrial-CK (CK-mito). the primary antibody was (sMt CK (c-18): sc15168 at 1:200 dilution, Santa Cruz) and secondary antibody Donkey anti-Goat HRP (Stratech 705-035-147) at 1:5000 dilution. Brain-CK (CK-B) was assessed with (CK-B (G-6): sc-374072 antibody, Santa Cruz). GAPDH was used as a loading control for protein normalization. Signals were quantified using Aida Image Analyzer (v4.22) and normalized to GAPDH.

### 2.5 Statistics

Results are presented as mean ± SEM. P < 0.05 was considered significant. Unpaired t-tests, one-way or two-way repeated measures ANOVA with multiple testing corrections were used as appropriate. Numbers of rats, hearts and myocytes used in each experiment are given in the relevant table and figure legends.

## 3 Results

### 3.1 Diastolic dysfunction *in vivo*

Consistent with previous reports, there was a significant increase in the heart: body weight and lung: body weight ratio in FAIL animals. The increase in heart weight was principally due to a significant increase in the RV: LV weight, indicating RV hypertrophy (Table 1).

**Fig. 1:**
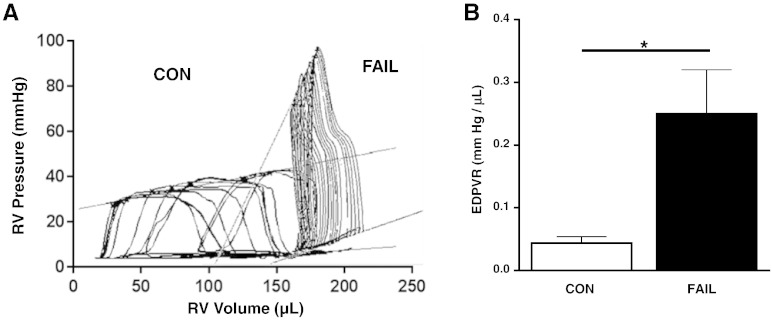
Measurement of EDPVR relationships. Final day *in vivo* RV hemodynamic measurements. A. Examples PV loops in CON and FAIL heart, lines indicate end diastolic pressure volume relationships (EDPVR) and end systolic pressure volume relationships. B. Mean EDPVR relationships. FAIL rats displayed significantly steeper EDPVR. N = 7 CON and 8 FAIL hearts. *P < 0.05.

**Fig. 2:**
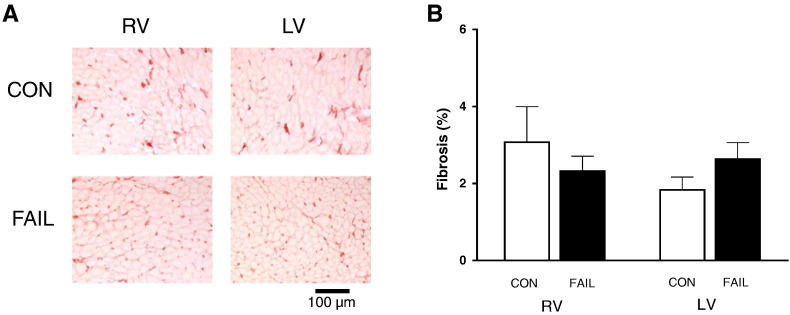
Measurement of collagen in CON and FAIL hearts. A. Example images of RV and LV sections stained with picrosirius red. Color thresholding was used to identify areas of collagen (red), the threshold value was optimized for each section and applied to both RV and LV. B. The area of collagen staining (%) was not different between the 4 regions, suggesting fibrosis was not increased at this stage of MCT-induced heart failure. N = 7 CON and 7 FAIL hearts.

*In vivo* measurement of RV pressure and volume showed a significant increase in systolic pressure (see Suppl. Table 1). PV loops (Fig. 1A) were used to calculate EDPVR which were significantly increased in FAIL hearts (Fig. 1B) suggesting diastolic dysfunction in the form of increased resistance to filling. Increased fibrosis could be a cause of diastolic dysfunction, however histological measurement of fibrosis revealed no significant differences between CON and FAIL hearts (Fig. 2).

### 3.2 Diastolic dysfunction in stretched single cardiac myocytes

**Fig. 3:**
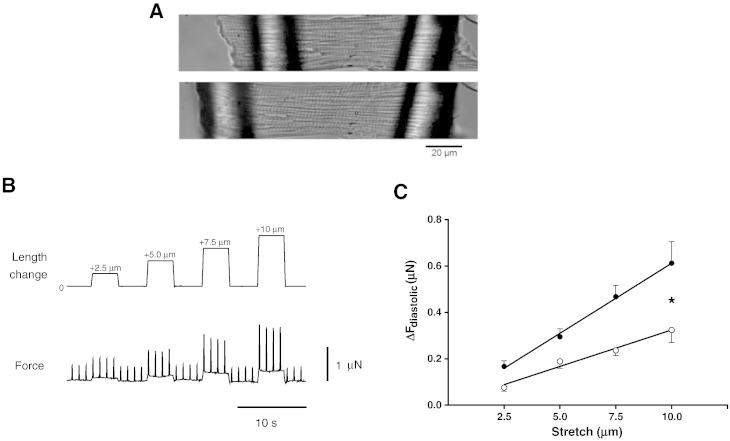
Diastolic force–length relationships in single RV CON and FAIL myocytes. A. Images of a single RV myocyte attached to glass fibers before and after a stretch that increased SL from 1.83 to 1.93 μm. B. Example of stepwise increases in cell length changes (*top*) separated by a return to resting length imposed on a CON cell and the corresponding change in force (*bottom*) during isometric contraction and relaxation. C. The difference in diastolic force between resting length and each stretch (ΔF_diastolic_) was used to construct an end-diastolic force–length relationship (EDFLR). The slope of the EDFLR was significantly steeper in FAIL (0.06 ± 0.01 μN/μm) compared to CON (CON 0.03 ± 0.01 μN/μm) (*P < 0.05). The distance between glass fibers at resting length was not different between CON and FAIL (CON 95.0 ± 4.9 μm, FAIL 95.6 ± 2.5, P = 0.90). N = 8 CON and 13 FAIL myocytes from 3 CON and 4 FAIL hearts.

The source of dysfunction might therefore originate within the myocytes. Indeed when single cardiac myocytes were attached to glass fibers and stretched (Fig. 3A, B) the resultant slope of the EDFLR (the cellular equivalent of the *in vivo* EDPVR) was significantly increased in RV FAIL myocytes (Fig. 3C). For consistency with previous reports, data are presented as the absolute change in force per unit distance [19]. Although FAIL myocytes tended to have a larger cross-sectional area (XSA) (RV FAIL 551 ± 60 μm^2^, RV CON 375 ± 29 μm^2^, P = 0.06), resting force normalized to XSA and % length change was still significantly greater in FAIL myocytes (CON 0.07 ± 0.01, FAIL 0.13 ± 0.03 mN·mm^− 2^. %^− 1^ length change, P < 0.05) indicating that larger XSA was not the sole cause of the steeper EDFLR in FAIL myocytes.

**Fig. 4:**
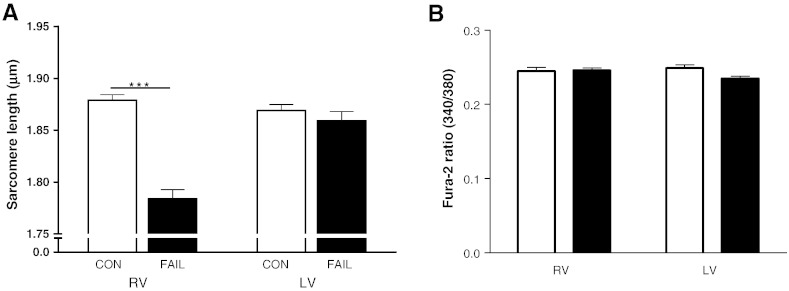
Diastolic sarcomere length and [Ca^2 +^]i in CON and FAIL myocytes. A. Resting SL in RV (N = 30 CON and 31 FAIL) and LV (N = 15 CON and 18 FAIL) myocytes from 6 CON and 11 FAIL hearts. Resting SL was significantly shorter in FAIL RV cells ***P < 0.001. B. There were no differences in diastolic [Ca^2 +^]i (indexed as Fura-2 340/380 ratio) between the 4 groups (RV, N = 21 CON and 23 FAIL; LV N = 21 CON and 24 FAIL myocytes) from 3 CON and 4 FAIL hearts.

**Fig. 5:**
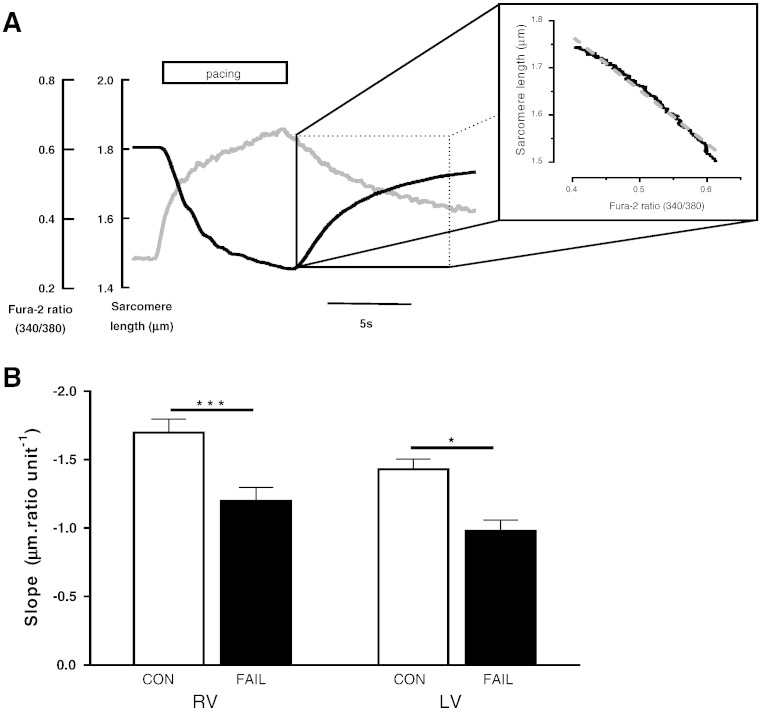
Estimation of myofilament Ca^2 +^ sensitivity in intact CON and FAIL myocytes. A. In intact cells (loaded with Fura-2 and in which SERCA is inhibited by thapsigargin) a near steady-state relationship between [Ca^2 +^]i and SL change was achieved during re-lengthening, following stimulation at 10 Hz in 5 mM Ca^2 +^-Tyrode at 20–23 °C. Inset demonstrates the linear relationship (r^2^ = 0.98) during relaxation. B. The mean slope of SL-340:380 ratio was less steep in both failing RV and LV cells compared to CON, suggesting decreased myofilament Ca^2 +^ sensitivity in FAIL myocytes. N = 22 CON and 17 FAIL; LV, N = 15 CON and 15 FAIL myocytes from 3 CON and 3 FAIL hearts. *P < 0.05, ***P < 0.001.

Resting SL of RV myocytes from FAIL hearts was significantly shorter than either LV myocytes from FAIL or RV myocytes from CON hearts (Fig. 4A). This shorter SL was not caused by raised diastolic [Ca^2 +^]i (fura-2 340:380 ratio was not different between groups, Fig. 4B). When intact myocytes re-lengthened following tetanization (Fig. 5A) the slope of the SL-fura-2 340:380 ratio was shallower in FAIL than CON myocytes (Fig. 5B). This slope is an index of myofilament Ca^2 +^ sensitivity, with a steeper slope indicating increased Ca^2 +^ sensitivity. The shallower slope in FAIL myocytes suggests that the shorter resting SL in RV FAIL myocytes was not caused by intrinsic higher myofilament Ca^2 +^ sensitivity.

### 3.3 Ca^2 +^-independent shortening of diastolic sarcomere length in failing myocytes

**Fig. 6:**
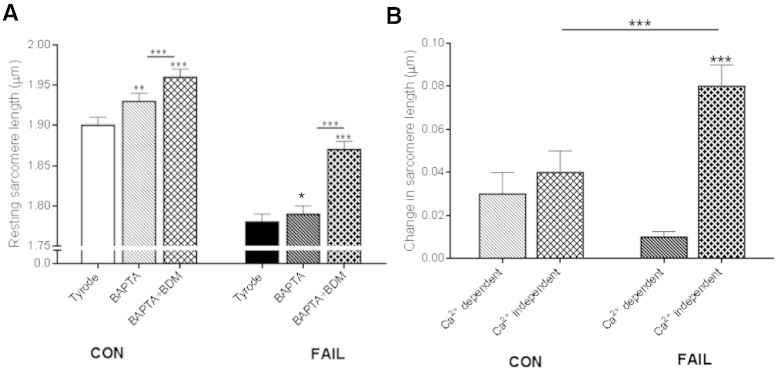
Ca^2 +^-dependent and -independent contribution to diastolic SL in CON and FAIL myocytes. A. Repeated measurement of diastolic SL in intact myocytes from the RV of CON and FAIL hearts in 1 mM Ca^2 +^ Tyrode solution (Tyrode), Tyrode plus BAPTA-AM (BAPTA) to buffer intracellular Ca^2 +^, and following further addition of 40 mM BDM (BAPTA + BDM) to inhibit myosin ATP-ase. There was a significant difference in SL in Tyrode between the 2 groups (CON > FAIL, not marked on figure for clarity). Within each of the 2 groups there was significant and progressive increase in SL following BAPTA and then BAPTA + BDM. B. Change in diastolic SL attributed to Ca^2 +^-dependent (BAPTA minus Tyrode) and Ca^2 +^-independent (BAPTA + BDM minus BAPTA) mechanisms. There was no difference in the Ca^2 +^-dependent change between the 2 groups. Ca^2 +^-independent change in FAIL myocytes was significantly greater than CON and Ca^2 +^-dependent changes in FAIL. N = 18 CON and 25 FAIL myocytes from 3 CON and 3 FAIL hearts, *P < 0.05, **P < 0.01, ***P < 0.001.

To investigate a possible Ca^2 +^-independent source of diastolic SL shortening in RV FAIL myocytes, resting SL was first measured in Tyrode's solution. When the same cells were exposed to the intracellular Ca^2 +^ buffer BAPTA-AM (100 μM) SL increased and increased further upon additional exposure to the myosin ATP-ase inhibitor BDM (40 mM) (Fig. 6A). The Ca^2 +^-dependent changes in SL (caused by BAPTA alone) were compared to the Ca^2 +^-independent change in SL (additional increase in SL caused by BDM). The Ca^2 +^-independent change in SL was significantly greater in FAIL myocytes compared to both the Ca^2 +^-independent change in SL in CON and to the Ca^2 +^-dependent change in SL in FAIL (Fig. 6B).

### 3.4 Creatine kinase expression and diastolic sarcomere length

**Fig. 7:**
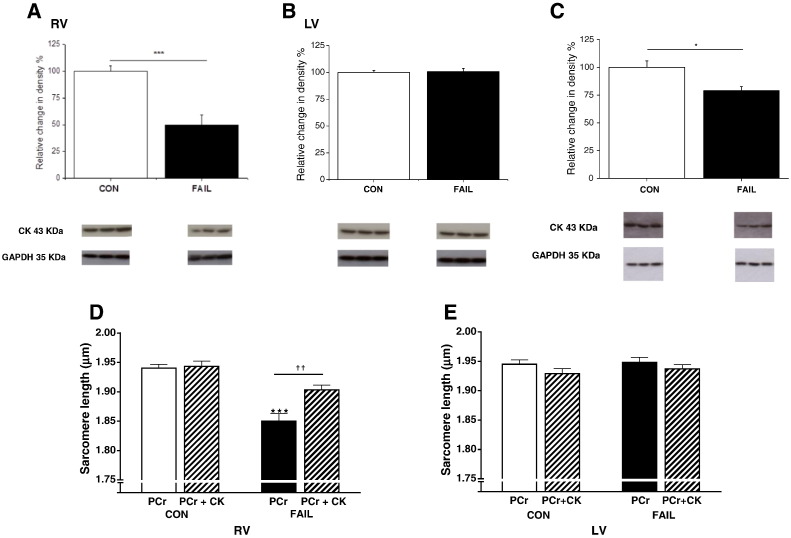
CK is decreased but exogenous CK lengthens SL in RV FAIL. Western blot analysis of CK-M in CON and FAIL tissue from A. RV and B. LV. In RV FAIL there was a significant reduction in CK-M compared to CON. In LV there were no differences between the groups. N = 6 RV and 3 LV in both groups, ***P < 0.001. C. There was a significant reduction in CK-mito in RV FAIL compared with RV CON. N = 7 in both groups, *P < 0.05. CK band intensity was normalized to the loading control GAPDH in A–C. Exogenous CK-M binds to the myofilaments of permeabilized cardiomyocytes and is functionally active. Saponin permeabilized cells were incubated in intracellular solution containing 10 mM phosphocreatine (PCr) or 10 mM PCr plus 4.4 mg/ml CK (PCr + CK). D. In RV permeabilized myocytes diastolic SL was shorter in FAIL myocytes in PCr solution compared to CON (***P < 0.001) and in solutions lacking PCr with or without exogenous bovine CK (Supplementary Fig. 1) compared to CON. Diastolic SL in RV FAIL myocytes was significantly increased by the addition of exogenous bovine CK + PCr (^††^P < 0.01 *vs* RV FAIL PCr), and was not significantly different to RV CON myocytes in the equivalent solution. E. There were no differences in SL between groups of LV myocytes. N = 36 myocytes in each group from 4 CON and 3 FAIL hearts.

Expression of CK-M was significantly reduced in RV FAIL (Fig. 7A) but not different between any LV groups (Fig. 7B). There was also a significant reduction in CK-mito in RV FAIL (Fig. 7C). We were unable to detect expression of CK-B in either CON or FAIL samples.

In the presence of 10 mM phosphocreatine, SL was significantly shorter in skinned RV FAIL myocytes than RV CON (as seen in intact cells). Exposure to 4.4 mg/ml exogenous CK caused no change in SL in CON cells but significantly increased SL in RV FAIL cells such that their SL was no longer significantly shorter than CON cells (Fig. 7D). This effect was dependent upon the presence of the substrate PCr because, exogenous CK-M did not lengthen SL in the absence of PCr (Suppl. Fig. 1). Resting SL was not different between the groups of LV myocytes and adding CK did not alter SL (Fig. 7E). The observations in Fig. 7 demonstrate a reversible link between CK levels and resting SL.

**Fig. 8:**
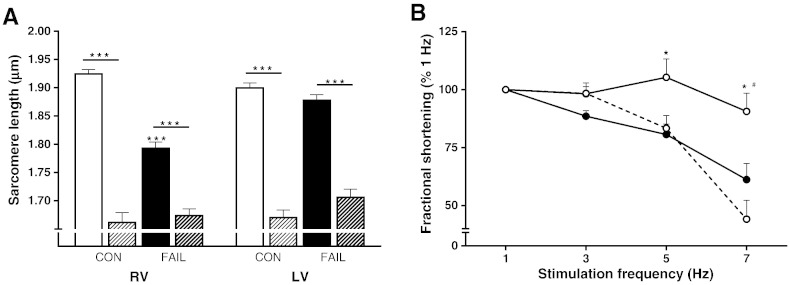
Inhibition of creatine kinase in RV CON myocytes mimics RV FAIL diastolic SL and contraction–frequency relationship. A. Endogenous CK was inhibited with 20 μM DNFB and the effect on resting SL measured. A. In the absence of DNFB, resting SL was significantly shorter in RV FAIL compared to CON (***P < 0.001). DNFB (hatched bars) significantly shortened resting SL in all groups of cells (***P < 0.001) such that SL was not different between groups of DNFB treated myocytes (N = 36 cells per group from 4 CON and 4 FAIL hearts). B. When stimulation frequency was increased in RV CON myocytes a relatively flat contraction amplitude–frequency relationship was seen (open symbols, solid line). This relationship was steeply negative in RV FAIL myocytes (closed symbols, solid line). The relationship in RV CON myocytes exposed to DNFB was also steeply negative (open symbols, dotted line). N = 23 CON, 28 FAIL, 25 CON + DNFB myocytes from 8 CON and 7 FAIL hearts, P < 0.05 *CON *vs* FAIL, ^#^CON + DNFB.

When intact cells were exposed to the CK inhibitor DNFB (20 μM) there was a significant shortening of SL in all cells (Fig. 8A) however the greatest effects were in LV cells and RV cells from CON hearts, that is, the cells with the highest levels of CK (see Fig. 7). Significantly shorter SL was also observed in permeabilized RV CON (− DNFB 1.94 ± 0.02 μm, + DNFB 1.88 ± 0.02 μm, P < 0.05) and LV CON myocytes (− DNFB 1.93 ± 0.01 μm, + DNFB 1.88 ± 0.01 μm, P < 0.001) exposed to DNFB, N = 10 myocytes in each group from 1 heart.

A systolic characteristic of RV FAIL cells is a steeply negative contraction–stimulation frequency relationship compared to CON cells [14] (Fig. 8B). CON myocytes exposed to DNFB displayed a steeply negative contraction–frequency relationship similar to FAIL myocytes (Fig. 8B).

## 4 Discussion

### 4.1 Animal model, PAH and RV failure

The increase in end systolic pressure in MCT-treated animals is evidence of PAH. The designation of our MCT-treated animals as heart failure is based on the appearance of previously validated external clinical signs *e.g.* [11–14]. The key clinical manifestations of RV failure have been stated as exercise limitations and fluid retention [20], consistent with this, MCT-treated animals showed a depressed myocyte contraction–frequency response (that is, a decreased response to increased demand) and increased wet lung: body weight, consistent with lung congestion. However the enhanced ESPVR and dP/dt max values (see Suppl. Table 1) do not support the presence of systolic failure. Intervention upon appearance of clinical signs was a condition of ethical approval and more likely occurs at the onset of heart failure than at end-stage failure when *in vivo* systolic dysfunction may become apparent.

### 4.2 Evidence for diastolic dysfunction at the level of the failing single myocyte

Reduction of CK activity has been described in human diseased myocardium [4,5] and has been linked to contractile dysfunction, reduction in contractile reserve [6,21–23] and increased mortality [5]. Consistent with these observations, we show decreased expression of the most abundant form of CK, in hearts from PAH animals with RV failure. We also present evidence for diastolic dysfunction in myocytes from failing hearts. This dysfunction was ameliorated by addition of exogenous CK and mimicked in healthy cells by pharmacological inhibition of CK. We therefore conclude that diminished CK activity is an important component of diastolic dysfunction induced by PAH.

Previous work has shown that diastolic function was preserved in MCT-induced hypertrophy prior to failure (30 mg/kg, MCT) [24]. We observed a steeper *in vivo* EDPVR in failing hearts but do not attribute this to increased fibrosis. At a similar stage of disease other MCT studies have not observed increase in fibrosis [25] while others have [12]. When increased fibrosis is present it could contribute to diastolic dysfunction by increasing stiffness and resistance to RV filling.

Investigation of the diastolic force-length relationship in single myocytes is a useful approach to isolate intracellular mechanisms from the influence of the extracellular matrix [26] and using this approach we saw a steeper EDFLR in failing myocytes. Diastolic dysfunction and increased passive stiffness in the myocytes of human sufferers of PAH have been linked to reduced phosphorylation of titin [26]. We did not investigate titin in our study but as rats express the shorter, stiffer N2B isoform of titin, any role of titin in rats is likely to be related to phosphorylation rather than isoform re-distribution. In contrast to Rain et al. [26] we observed shorter resting SL and endogenous actin-myosin activation. These differences may be related to species differences and our use of intact or saponin permeabilized myocytes, compared to pre-frozen and triton-skinned myocytes from end-stage heart failure patients [26].

### 4.3 Creatine kinase and mechanisms determining diastolic sarcomere length

We observed significant reduction of the 2 major CK isoforms (CK-M and CK-mito) in RV FAIL hearts. Commonly described regulators of diastolic SL are diastolic [Ca^2 +^]i and myofilament Ca^2 +^ sensitivity, however we saw no evidence of an increase in either of these parameters in failing myocytes. We do provide evidence that the reduction in diastolic SL in failing cells occurred predominantly through a Ca^2 +^-independent (BDM-sensitive) rather than a Ca^2 +^-dependent (BAPTA-sensitive) mechanism. Importantly, we show that reduced diastolic SL was improved by addition of exogenous CK in the presence of PCr, suggesting a requirement for functional CK rather than just its physical presence, and mimicked in healthy cells by pharmacological inhibition of CK. Regulation of SL or passive tension by increased ADP is well-documented [27] as is an association between reduced CK activity and increased ADP [28]. A local accumulation of ADP can occur if myofibrillar CK activity is sufficiently impaired. As a consequence, rigor-like and slowly cycling, cross-bridges can form which leads to Ca^2 +^-independent force production [29,30]. There is evidence that other metabolic pathways are also compromised in this condition, for example, reduced creatine content [24], decreased CK-mito activity, mitochondrial dysfunction and increased glycolysis [31–33].

The steeper contraction–frequency relationship in RV FAIL myocytes is a cellular manifestation of the inability of failing hearts to respond to increased demand (the characteristic that underpins the NYHA classification of heart failure). The observation that inhibition of CK in CON myocytes mimics the contraction-frequency relationship of RV FAIL myocytes supports a central role of metabolic dysfunction in this key characteristic of heart failure [21].

Interestingly single isoform (MCK^−/−^, mitochondrial CK^−/−^ [34]) and double (CK^−/−^ [35,36]) CK knockout mice show a mild cardiac phenotype, demonstrating resilience to a non-functioning CK system, some of which may be strain or sex-dependent [36] or be related to compensatory mechanisms such as cytostructural changes to reduce intracellular diffusion distances [37]. Creatine-deficiency through knockout of the creatine transporter [38] or biosynthetic pathway (GAMT^−/−^ [39]) is well tolerated, with mice able to exercise and survive experimental myocardial infarction [40]. The GAMT^−/−^ mice were reported to have smaller hearts and lower pressures [40]. In the MCT model the combination of reduced CK enzyme and creatine substrate [24] in hearts that are hypertrophic, hypertensive and inflammatory [41] may contribute to the differences between findings in the MCT and KO models.

### 4.4 Study limitations

We did not measure CK activity and there is a possible mismatch between protein levels and activity, however in a pressure-overload model of LV failure [42] reported similar levels in the reduction of CK-mito protein and CK-mito activity, supportive of a link between protein and activity levels. Our inhibitor of CK may have off target effects such as the modification of sulfhydryl groups that could affect Ca^2 +^-handling channels such as RYR2 [43] but we do show that in permeabilized myocytes, where [Ca^2 +^]i is controlled, DNFB still reduced resting SL. It is difficult to directly equate our measures of SL in isolated myocytes to those seen *in vivo* during a cardiac cycle. *In vivo* SL is still a subject of research, but *in situ* SL measured in arrested mouse hearts was found to be close to ~ 1.95 μm, similar to our CON SL of ~ 1.90 μm [44], although the mechanical loading conditions *in vivo* and in isolated myocytes is different.

### 4.5 Conclusions

Currently, PAH treatment is focused on the pulmonary vasculature, based on the rationale that the reversal of pulmonary hypertension will decrease the stresses on the heart that lead to RV hypertrophy and failure. However, at present heart failure can be treated but not cured. Therefore the manifestation of RV dysfunction cannot be disregarded. Novel therapies targeting both pulmonary vascular constriction and RV failure should be considered. Our data suggest that cardiac energetics would be a valid potential target for therapeutic intervention in PAH.

## Funding sources

University of Leeds PhD scholarship to EDF; Emma and Leslie Reid PhD scholarship to DB; British Heart Foundation grant PG/13/3/29924. CVON grant (2011-11-ARENA). Travel grants to EDF from the Physiological Society and the Boehringer Ingelheim Fonds.

## Disclosures

MH is an employee of IonOptix LLC.

## Figures and Tables

**Table 1:** Animal and organ weights. Final day parameters for saline treated (CON) and 60 mg/kg MCT (FAIL) animals. Right ventricular free wall (RV), left ventricular free wall (LV). *P < 0.05, ***P < 0.001 *vs* CON.

	CON (N = 38)	FAIL (N = 43)
Body weight (g)	323 ± 3.4	267 ± 3.6***
Heart weight (g)	1.20 ± 0.02	1.42 ± 0.04***
RV weight (g)	0.24 ± 0.01	0.38 ± 0.02***
LV weight (g)	0.58 ± 0.02	0.52 ± 0.02*
Lung weight (g)	1.87 ± 0.11	2.56 ± 0.09***
Liver weight (g)	12.85 ± 0.22	10.82 ± 0.29***
Heart weight/body weight (mg/g)	3.64 ± 0.13	5.33 ± 0.14***
RV weight/LV weight (mg/mg)	0.42 ± 0.02	0.77 ± 0.04***
Lung weight/body weight (mg/g)	5.63 ± 0.34	9.66 ± 0.37***
Liver weight/body weight (mg/g)	38.8 ± 1.24	40.28 ± 0.74
