# Supplementary material for: Decreased creatine kinase is linked to diastolic dysfunction in rats with right heart failure induced by pulmonary artery hypertension
Source: J Mol Cell Cardiol. 2015 Sep;86:1–8. doi: 10.1016/j.yjmcc.2015.06.016 (PMC4564291; doi:10.1016/j.yjmcc.2015.06.016)
Supplement: Supplementary file 1 — Supplementary material. [file mmc1.pdf]

Decreased creatine kinase is linked to diastolic dysfunction in rats with right heart failure induced by pulmonary artery hypertension

Ewan D Fowler, David Benoist, Mark J Drinkhill, Rachel Stones, Michiel Helmes, Rob CI Wüst, Ger JM Stienen, Derek S Steele, Ed White

Online Supplementary material: Expanded Methods

Male Wistar rats (200g) received a single intraperitoneal injection of 60 mg/kg MCT to induce RV failure (FAIL) or an equivalent volume of saline as control (CON). Rats were killed upon showing clinical signs of heart failure (weight loss on consecutive days, dyspnea, cold extremities, lethargy) or on equivalent days for CON animals e.g. [1-3]. Experiments were conducted in accord with Health Research Extension Act (public law 99-158, 1985 "Animals in Research" and in accordance with the Directive 2010/63/EU of the European Parliament UK Home Office regulations and local ethical approval.

In vivo haemodynamics

In vivo haemodynamic measurements were made in anesthetized rats. Rats were intubated and mechanically ventilated with 1.5% isoflurane mixed with O_2. The chest was opened and a Millar conductance catheter passed through the RV free wall into the RV cavity to simultaneously measure pressure and volume. Ejection fractions were calculated from max. systolic and min. diastolic volumes. Pressure-volume (PV) loops were recorded and the slope of the end-diastolic pressure-volume relationship (EDPVR) measured by occlusion of the ascending vena cava. A cannula was inserted into the left jugular vein to inject a bolus (20 µl) of hypertonic saline (15%) in order to determine the parallel conductance of

surrounding tissue. Prior to cardiac catheterization the Millar device was calibrated for pressure using a mercury manometer. At the end of the experiment fresh heparinized blood was collected from the animal and used to fill a cuvette (Millar P/N 910-1048) to calibrate for volume. Volumes were calibrated at the end of the procedure by measuring conductance in known blood volumes and parallel conductance subtraction. Alpha changes throughout the cardiac cycle as the ventricles fill and empty. In our calibration procedure we have assumed alpha to be 1 for both CON and FAIL rats. Although there is some nonlinearity of the volume signal because of the positioning of the ring electrodes along the conductance catheter, in species with relatively small hearts such as the mouse and rat where the distance is not great, this nonlinearity is considered to be less problematic (see Pacher et al, [4]) with good linearity being observed with Doppler measurements (see Georgakopoulos et al [5]). Differences in alpha between the CON and FAIL hearts would introduce error in the comparison of volumes between the two groups.

Histological assessment of collagen

Isolated hearts were rapidly frozen in liquid N_2-cooled isopentane and stored at -80°C. 10 µm thick cryosections were cut through the short axis of hearts and attached to poly-L-lysine coated microscope slides, fixed in 4% formaldehyde in PBS and stained with picrosirius red for 90 min. In each heart a 0.15 mm^2 area from the posterior, mid and anterior aspect of each ventricular free wall were taken from a section mid-way between base and apex were imaged using a Nikon Eclipse E600 light microscope with a 20x Nikon Plan Fluor objective. Red collagen staining was identified using a colour thresholding algorithm in ImageJ (National Institute of Health, Bethesda, USA). The threshold was optimised for each section to account for variable staining intensity but was kept constant for all regions of a particular section. For every ventricle the average of the 3 values was

calculated. The experimenter was blinded to the subject group (CON or FAIL) during image acquisition and analysis.

Isolated myocyte studies

Myocytes were isolated according to McCrossan *et al* [6]. Hearts were Langendorff-perfused with enzyme solution containing 1 mg/ml collagenase and 0.1 mg/ml protease for 7 min then the RV and LV free wall were dissected and isolated myocytes obtained by shaking in enzyme solution. Cells were stored at room temperature for up to 8 h in Tyrode's solution containing, in mM: 137 NaCl, 5.4 KCl, 0.33 NaH_2PO_4, 0.5 $MgCl_2$, 5 HEPES, 5.6 glucose, 1$CaCl_2$, pH 7.4 with 5N NaOH.

Intracellular Ca^{2+} ($[Ca^{2+}]i$) was monitored using the ratiometric Ca^{2+} dye fura-2 -AM (Invitrogen, UK) [6]. Cells were incubated with fura-2 -AM (final concentration 3.1 μM) for 10 min, then washed and left for 30 min to allow full de-esterification. Fura-2 loaded cells were alternately illuminated with 340 and 380 nm light and the ratio of emitted light collected at 510 nm used to detect changes in $[Ca^{2+}]i$. Simultaneous recordings of SL and fura-2 signal were acquired using Ion Wizard software (Ionoptix, Milton, MA, USA). Myocytes were field stimulated via external platinum electrodes at the desired frequency. Unless otherwise stated, experiments were carried out at 37 ºC.

In experiments investigating the control of resting SL, Tyrode solution was modified by inclusion of the intracellular Ca^{2+} buffer BAPTA-AM (100 μM) or BAPTA-AM plus the cycling myosin ATP-ase inhibitor 2,3-butanedione monoxime (BDM; 40 mM, Sigma, UK). To inhibit all forms of CK, the irreversible agent 2, 4-dinitro-1-fluorobenzene (DNFB; 20 μM, Sigma, UK) was added for 10 min. Cells were then washed 3 times in 1 mM Ca^{2+} Tyrode solution.

Saponin (0.01 mg/ml) was used to permeabilize myocytes in an intracellular-like solution containing, in mM: 130 K^+, 30 Na^+, 135.7 Cl^-, 25 HEPES, 5.7 Mg^{2+}, 5 ATP, 10 PCr, 0.5 EGTA, 0.01% BSA, pH adjusted to 7.0 with 8N KOH, free Mg^{2+} was 1 mM [7]. In experiments testing the role of CK in controlling resting SL, the muscle isoform (MM)-CK from bovine heart muscle (Sigma, UK) was added to the solution that perfused the skinned myocytes, at a concentration of 4.4 mg/ml (total activity 250 U/ml). Cells were incubated in intracellular solution with or without exogenous CK for 30 min before SL was measured at 20-23°C.

Intact myocyte myofilament Ca^{2+} sensitivity

The method of Hongo *et al* [8] was used to measure myofilament Ca^{2+} sensitivity in intact myocytes. Fura-2 -AM loaded cells were incubated for 15 min with the irreversible SERCA inhibitor thapsigargin (1 μM), then field stimulated for 10 s at 10 Hz at 20-23°C in 5 mM Ca^{2+} Tyrode solution. This caused a sustained rise in $[Ca^{2+}]i$ and a tetanus-like contraction. When pacing was stopped $[Ca^{2+}]i$ was slowly removed (primarily through the Na-Ca exchanger), during this time the SL increased in near equilibrium with Ca^{2+} removal, resulting in a linear relationship (r^2 = 0.92-0.99) whose slope is an index of Ca^{2+} sensitivity in intact cells.

Single cell force-length relationship

Mechanical manipulation of myocytes was performed with a MyoStretcher (Ionoptix, Milton, USA). Myocytes were placed in the bath of an inverted microscope in 1.8 mM Ca^{2+} Tyrode solution at 37°C and field stimulated at 1 Hz. Myocytes were attached to stiff glass fibers using MyoTak™ glue (IonOptix) [9]. One fiber was connected to a high sensitivity optical force transducer (OptiForce, IonOptix), the other fiber to a computer controlled piezo length translator (Mad City labs, Madison, USA) [10]. Cells were stretched between

2.5 and 10 µm while isometric force transients were recorded. The end diastolic force-length relationship (EDFLR), the cellular equivalent of the *in vivo* EDPVR, was calculated by measuring the change in diastolic force (µN) between rest and stretch. Myocyte cross-sectional area (XSA) was estimated from cell width assuming a width:depth ratio of 1.44 [11]. Modifying the isolation procedure by omitting protease from the enzyme mix improved the viability of myocytes when attached to glass fibers. Attached myocytes produced stable isometric contractions for >15 min.

Western blot analysis of CK protein

RV and LV epicardial tissue isolated from CON and FAIL animals were prepared for Western Blot analysis of CK as follows. Isolated hearts were dissected in an ice cold Ca^{2+} free solution (130 NaCl, 5.4 KCl, 1.4 $MgCl_2$, 0.4 NaH_2PO_4, 0.75 $CaCl_2$, 5 HEPES, 10 glucose, 20 taurine and 10 creatine, pH 7.4 with NaOH). RV and LV tissue was weighed and finely minced in an appropriate amount of cold sample buffer containing the following, TRIS/HCl (pH6.8) 62.5 mM, Glycerol 10%, SDS 2%, and supplemented with Complete Protease Inhibitor (CPI, 40 µl/ml, Roche) and Halt Phosphatase Inhibitor cocktail (HPIC, 10 µl/ml, Thermo Scientific) per 0.1 g of tissue. The sample was then homogenised for 4 x 10 s on ice and decanted into Eppendorfs and centrifuged for 10 min. at 16,000 g at 15 °C. The resulting pellet was discarded and the supernatant saved and stored at -20 °C for subsequent protein concentration determination by bicinchoninic acid assay and Western Blotting. 20 µg of total protein from RV and LV epicardium were separated on 4-20% Mini Protean TGX resolving gels (Bio-Rad) under reducing conditions (5% 2-mercaptoethanol) followed by semi-dry transfer of the proteins onto PVDF membranes and probing by antibodies for CK-M primary antibodies, (MM-2) sc-69848, 1:500, Santa Cruz Biotech; anti-GAPDH, G9545, 1:100,000, Sigma USA) overnight at 4°C. Anti-GAPDH

was used as a loading control for protein normalization. Signals were visualized with goat-anti-mouse (1:10,000 for CK-M) and goat anti-rabbit (1:5000 for GAPDH) secondary antibodies (Jackson Immuno Research Laboratories Inc., USA) following enhanced chemiluminescence (Supersignal West Pico Chemiluminescent Substrate, Pierce, USA). Non-saturated signals were quantified using Aida Image Analyzer (v4.22) and normalized to the band density of GAPDH. For CK-mito the primary antibody was (sMt CK (c-18): sc15168 at 1:200 dilution, Santa Cruz) and secondary antibody Donkey Anti-Goat HRP(Stratech 705-035-147) at 1:5000 dilution. CK-brain was assessed with (CK-B (G-6): sc-374072 antibody, Santa Cruz Biotech). Despite a 2.5 fold increase in protein loading compared to CK-M no detectable CK-B signal was seen in either CON or FAIL hearts.

Results

Haemodynamic parameters are presented in Suppl. Table1. Compared to CON hearts, FAIL hearts showed significant increases in end systolic pressure, consistent with PAH, and EDPVR, consistent with diastolic dysfunction, also increased ESPVR and dP/dt which is inconsistent with systolic heart failure (see Discussion).

We tested whether exogenous CK-M (4.4 mg/ml) affected resting SL in saponin permeabilized cardiomyocytes when physically present but not functionally active, by removing the substrate, phosphocreatine (PCr). Selective addition of endogenous CK in the presence or absence of PCr revealed that endogenous CK only lengthened SL in FAIL RV myocytes in the presence of PCr (Suppl. Fig.1).

	CON (N=10)	FAIL (N=12)
End-systolic pressure (mmHg)	26.0 ± 2.7	77.6 ± 5.6***
End-diastolic pressure (mmHg)	4.4 ± 0.7	8.0 ± 1.0*
dP/dt max (mmHg/s)	2441 ± 265	4506 ± 547***
dP/dt min (mmHg/s)	-2521 ± 281	-3990 ± 618 p= 0.05
Tau (ms)	7.8 ± 0.7	12.0 ± 0.8***
End-systolic volume (µL)	27.0 ± 8.7	64.4 ± 11.1*
End-diastolic volume (µL)	61.5 ± 14.5	91.4 ± 13.0
Ejection fraction (%)	69.8 ± 6.8	41.0 ± 3.0***
Arterial elastance (Ea) (mmHg/µL)	0.99 ± 0.29	2.5 ± 0.38**
End systolic elastance (ESPVR) (mmHg/µL)	0.38 ± 0.15 (N=7)	2.55 ± 0.67* (N=8)
End Diastolic elastance (EDPVR) (mmHg/µL)	0.04 ± 0.01 (N=7)	0.25 ± 0.07* (N=8)

Supplementary Table 1. Pressure volume parameters. Final day haemodynamic parameters for saline treated (CON) and 60 mg/kg MCT (FAIL) animals. * $P < 0.05$, ** $P < 0.01$, *** $P < 0.001$ vs CON.

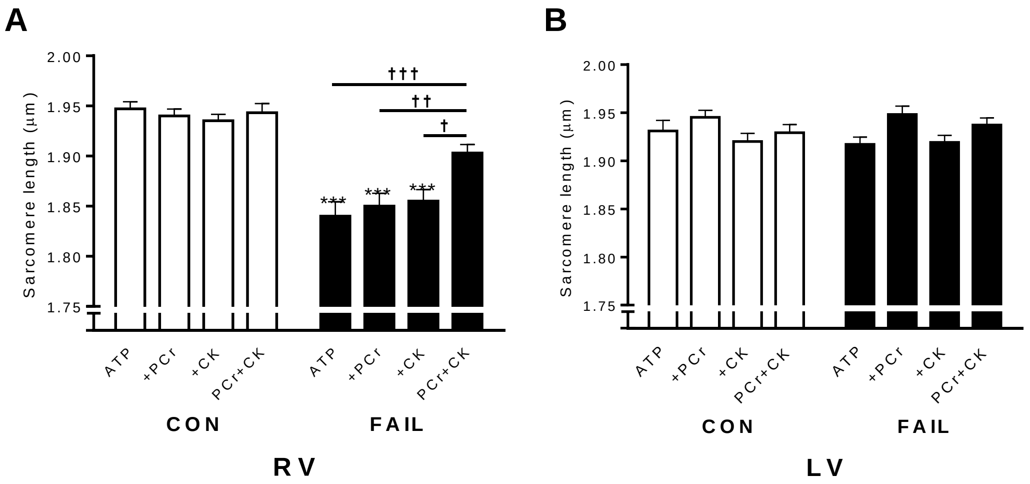

Supplementary Figure 1. Exogenous creatine kinase (CK) requires its substrate, phosphocreatine (PCr), to increase diastolic SL in saponin permeabilized, RV FAIL myocytes. A RV myocytes or **B** LV myocytes were exposed to intracellular-like solution in the absence of both PCr and exogenous CK (ATP); the presence of either PCr or CK (+PCr, +CK) or the presence of both (PCr+CK). Sarcomere length of RV FAIL cells was significantly shorter than equivalent CON groups with the exception of the (PCr+CK) groups. FAIL RV in the presence of PCr+CK had significantly longer SL than other FAIL RV groups. There were no significant differences between LV groups. *** $P < 0.001$ vs CON in equivalent solution; † $P<0.05$, †† $P<0.01$, ††† $P<0.001$ vs RV FAIL PCr+CK. N = 36 myocytes in each group from N = 4 CON and 3 FAIL hearts.

Reference List

(1) Benoist D, Stones R, Drinkhill M, Bernus O, White E: Arrhythmogenic substrate in hearts of rats with monocrotaline-induced pulmonary hypertension and right ventricular hypertrophy. *Am J Physiol Heart Circ Physiol* 2011;300:H2230-H2237.

(2) Benoist D, Stones R, Drinkhill MJ et al.: Cardiac arrhythmia mechanisms in rats with heart failure induced by pulmonary hypertension. *Am J Physiol Heart Circ Physiol* 2012;302:H2381-H2395.

(3) Stones R, Benoist D, Peckham M, White E: Microtubule proliferation in right ventricular myocytes of rats with monocrotaline-induced pulmonary hypertension. *J Mol Cell Cardiol* 2013;56:91-96.

(4) Pacher P, Nagayama T, Mukhopadhyay P, Batkai S, Kass DA: Measurement of cardiac function using pressure-volume conductance catheter technique in mice and rats. *Nat Protoc* 2008;3:1422-1434.

(5) Georgakopoulos D, Kass DA: Estimation of parallel conductance by dual-frequency conductance catheter in mice. *Am J Physiol Heart Circ Physiol* 2000;279:H443-H450.

(6) McCrossan ZA, Billeter R, White E: Transmural changes in size, contractile and electrical properties of SHR left ventricular myocytes during compensated hypertrophy. *Cardiovasc Res* 2004;63:283-292.

(7) Steele DS, Smith GL: Effects of 2,3-butanedione monoxime on sarcoplasmic reticulum of saponin-treated rat cardiac muscle. *Am J Physiol* 1993;265:H1493-H1500.

(8) Hongo K, Kusakari Y, Konishi M, Kurihara S, Mochizuki S: Estimation of myofibrillar responsiveness to Ca2+ in isolated rat ventricular myocytes. *Pflugers Arch* 1998;436:639-645.

(9) Prosser BL, Ward CW, Lederer WJ: X-ROS signalling is enhanced and graded by cyclic cardiomyocyte stretch. *Cardiovasc Res* 2013;98:307-314.

(10) Iribe G, Helmes M, Kohl P: Force-length relations in isolated intact cardiomyocytes subjected to dynamic changes in mechanical load. *Am J Physiol Heart Circ Physiol* 2007;292:H1487-H1497.

(11) Natali AJ, Wilson LA, Peckham M, Turner DL, Harrison SM, White E: Different regional effects of voluntary exercise on the mechanical and electrical properties of rat ventricular myocytes. *J Physiol* 2002;541:863-875.
